# A non-invasive feather-based methodology for the detection of blood parasites (Haemosporida)

**DOI:** 10.1038/s41598-023-43932-y

**Published:** 2023-10-04

**Authors:** Merit González-Olvera, Arturo Hernandez-Colina, Julian Chantrey, Simon Allen, Javier Lopez, Matthew Baylis

**Affiliations:** 1 Institute of Infection, Veterinary and Ecological Sciences, University of Liverpool, Ic2 Liverpool Science Park, 146 Brownlow Hill, Liverpool, L3 5RF UK; 2 North of England Zoological Society (Chester Zoo), Caughall Road, Chester, CH2 1LH UK; 3 Gower Bird Hospital, Sandy Lane, Pennard, Swansea, SA3 2EW UK

**Keywords:** Molecular biology, Zoology, Biological techniques

## Abstract

Blood parasite (haemosporidian) infections are conventionally detected using blood samples; this implies capturing and handling birds to obtain them, which induces stress and causes pain. Feathers have blood vessels, and some blood could be preserved in the feather’s shaft after moulting. We used feather DNA for detecting haemosporidians by PCR testing in diverse scenarios. First, haemosporidian DNA was detected in feathers from carcasses of infected birds, proving the feasibility of the approach. Storage temperature affected DNA recovery, with maximum retrieval and haemosporidian detection at the lowest temperature (− 20 °C). All feather types from infected birds kept at optimal conditions yielded haemosporidian DNA. Parasite detection by PCR was correlated with DNA yield, which was significantly higher in heavier birds, flight feathers, and more feathers per pool. Lastly, haemosporidians were detected employing feathers moulted from wild and captive birds to estimate infection prevalence. We show for the first time that using blood from feather shafts for haemosporidian detection can be an advantageous and less invasive alternative to blood sampling if feathers are optimally preserved. This method could contribute to uncovering haemosporidian infections in endangered and elusive birds, and it might facilitate routine screening in captive birds, thereby improving infection detection, prevention, and control.

## Introduction

Haemosporidian parasites are a diverse group of vector-borne organisms that infect the blood and organs of their hosts at some stage of their development^1^. Clinical impacts of the most common avian haemosporidians (*Haemoproteus* spp., *Plasmodium* spp. and *Leucocytozoon* spp.)^2^ range from asymptomatic infection to severe illness^3–5^ and even death in some cases^6–11^; thus, affecting the health, welfare, and conservation of susceptible birds^6,7,10–13^. Conventional methods for detection of blood parasites in living birds include Polymerase Chain Reaction (PCR)^14^, microscopy^15^ and, less commonly, Enzyme-Linked Immunosorbent Assay (ELISA)^16,17^. These methods require a fresh blood sample, which implies restraining and handling birds. Stress induced from bird handling may affect breeding success and feeding^17^, aggravate underlying diseases or lead to sudden death^18,19^. The collection of a blood sample therefore requires consideration, weighing the associated costs versus its benefits. Hence, it is often decided not to take the sample^6^, and blood samples are opportunistically obtained when birds are in hand for other veterinary or research requirements^20^. This interferes with prompt disease diagnosis and start of early therapy^6^.

Feathers receive nutrients from a single axial artery located in the mesenchymal pulp; during feather development, a narrow channel forms in the calamus wall through which the mesenchymal pulp crosses. Once the feather develops, the mesenchymal pulp is reabsorbed from the calamus, leaving only keratinized pulp caps and a remnant of the axial artery, which is often visible as a blood clot on the superior umbilicus^21^. Eventually, the developed feather is moulted, and residual blood cells remain inside the feather shaft, protected from degradation by microorganisms, hydrolysis, solar radiation, and temperature variation^22^.

The use of blood contained in feathers could provide diagnostic material in cases where stressing the birds for conventional sampling is a concern, and additionally, it may be an effective way to obtain biological material from endangered or elusive species in the wild^21,23^, minimising disturbance or reducing risk of harm to individuals. Furthermore, feather collection does not require intensive training nor laboratory materials, and feathers are easy and cheap to store^24^.

Compared to other DNA sources, feathers may present lower quality and quantity of extracted DNA, leading to poor reliability of results^23^. Nevertheless, recent studies have achieved similar DNA yields to those seen on blood and tissue (1–100 ng/µl)^22,23^. These results are possibly related to the use of feather types and parts that produce different DNA yields; for instance, the use of remiges (flight feathers of the wing) and rectrices (flight feathers of the tail) instead of covert feathers (feathers that cover other feathers)^21,22,25^ or the superior umbilicus^21,22^ instead of the full calamus^22^, or its basal tip^21,23,25,26^. Likewise, the use of modern commercial DNA extraction kits^23,24^, as opposed to older protocols (e.g., phenol–chloroform)^25,26^ have enhanced the DNA yields. Lastly, it has been noted that shed feathers exposed to the environment for several months have only a 50% success in genotyping bird populations^23^, whereas feathers collected soon after shedding render higher amplification outputs^22^.

Feathers have been used in multiple genetic tests^22–25,27^; but they have been used for molecular diagnosis for two viral diseases only, Psittacine beak and feather disease^28^ and Marek´s disease^29^, both of which directly affect the feathers. The most common avian blood parasites (*Haemoproteus* spp., *Plasmodium* spp. and *Leucocytozoon* spp.) are not known to directly affect feathers or to deposit in feather capillaries; although, in some cases, ruffled feathers have been observed on infected birds^2,6,30^ and it has been found that these parasites may indirectly affect feather development through nutritional resource reallocation^31^. Here we hypothesise that haemosporidian DNA detection may be possible from the remnant blood retained inside the feather´s shaft during its development. This work aimed to evaluate the feasibility and reliability of using feathers for detecting haemosporidian infections and establishing guidelines for the use of feather samples as a diagnostic tool under diverse settings.

## General materials and methods

The general procedure consisted of collecting feathers, extracting DNA from them, and using that extract for PCR testing of haemosporidians. Since this is a new methodology, we first aimed at demonstrating its feasibility, then explored relevant aspects for its application in field investigations, such as sample preservation conditions, the type of sample to use and the adequate concentration of DNA; lastly, we tested it in surveillance situations. Therefore, we analysed five datasets to answer fieldwork related questions independently: (1) Can feathers be used to detect haemosporidian DNA in communities of wild birds? (2) What is the environmental influence on feathers’ DNA preservation and parasite detection success? (3) How do the number and type of feathers and bird weight influence DNA recovery and parasite testing? (4) Is it possible to use this method for passive surveillance in net-captured wild birds? and (5) Can this method be used for parasite surveillance in a captive population of wild birds? Each question is presented in its own section and a summary of the datasets per section can be found in Table 1. As the objectives of questions 1–3 were set to compare the use of feathers to conventional procedures, only haemosporidian-positive birds, confirmed by organ or blood sample testing, were used, while for questions 4 and 5, bird populations with unknown prevalence were screened to ascertain best practices for surveillance.

**Table 1:** Question, aims and main data features of this study.

Section	Aim regarding the use of feather samples	Bird group or species	Type of birds	Status	Samples	Diagnostic test	Parasite genus	Sequencing type
1	Detection of haemosporidians in different bird species	Multiple	Fl	Dead	Br Li F	PCR	H, P, L	F
2	Environmental influence on DNA recovery and parasite detection	Red-breasted goose	C	Dead	Bl Br Li F	PCR, Bs	P	F
3	Influence of number and type of feathers and bird weight on DNA recovery and parasite detection	Multiple	Fl	Dead	Bl Br Li F	PCR, Bs	H, P, L	F*
4	Detection of haemosporidians in a free-living bird population	Eurasian blackbird	Fl	Alive	F	PCR	H, P, L	F
5	Detection of haemosporidians in a captive bird population	Sumatran laughingthrush	C	Alive	F	PCR	L	F*

Fl: Free-living; C: Captive; Br: Brain; Li: Liver; F: Feathers; Bl: Blood; Bs: Blood smear; H: *Haemoproteus* spp. P: *Plasmodium* spp. ; L: *Leucocytozoon* spp.; F: Forward.

*Some samples were sequenced in both directions.

### Feather and organ collection from carcasses

Prior to post-mortem examination (PME), body weight was recorded and covert feathers (CF) from the breast and back, as well as primary and secondary remiges and rectrices (RF) were plucked from the bird carcasses; feathers were stored in plastic bags at − 20 °C. At PME, brain and liver samples were taken and stored at − 20 °C.

### DNA extraction

DNA extraction from organ and blood samples was conducted with a DNeasy Blood and Tissue kit (© QIAGEN), following the manufacturer´s instructions. DNA from CF was extracted by pooling the calamus of up to 20 feathers from the same individual, unless stated otherwise. For RF, the calamus of one feather was cut longitudinally and its content used for extraction (Supplementary Figure S1). Each feather was inspected so that if skin was still attached, it was removed using fine tweezers. Feather DNA extraction was done with an E.Z.N.A.^®^ Tissue DNA Kit (OMEGA Bio-Tek, Norcross, Georgia, USA) as this kit provided better DNA yields in preliminary feather extractions compared to the first one. The manufacturer´s instructions were followed except that, after adding the first lysis buffer and proteinase K, the sample was left incubating overnight at 55 °C in a shaking incubator, and the samples were eluted with 40 µl of elution buffer. For RF, the optional step of centrifuging and transferring the supernatant to a 1.5 ml tube after the incubation was done, as it was observed that these feathers tend to block the columns. Nucleotide concentration of the extractions was measured using a Nanodrop 1000 spectrophotometer (Thermo Fisher Scientific Inc; Wilmington, USA).

### Polymerase chain reaction

DNA extracted from bird organs, blood samples and feathers, was tested by nested PCR based on the method described by Hellgren^32^, which can detect *Plasmodium* spp., *Haemoproteus* spp., and *Leucocytozoon* spp. and consists of two amplification steps. For the first step, each reaction included 1 µl of DNA template, 1 µl of forward (HaemNF1) and reverse (HaemNR3) primers at a 10 µM, 10 µl of My Taq™ Red Mix (Bioline Reagents Ltd), 1 µl of BSA (Bovine Serum Albumin) and 6 µl of nuclease free water, to reach a final volume of 20 µl. The PCR profile was 22 cycles at 94 °C for 3 min, 94 °C for 30 s, 50 °C for 30 s, 72 °C for 45 s, followed by an extension at 72 °C for 10 min. The second step was performed using 2 µl of PCR product from the previous reaction, 1 µl of forward and reverse primers at a 10 µM, 10 µl of My Taq™ Red Mix (Bioline Reagents Ltd), 1 µl of BSA and 5 µl of nuclease free water, to reach a final volume of 20 µl. The profile for the second part was 36 cycles at 94 °C for 3 min, 94 °C for 30 s, 50 °C for 30 s, 72 °C for 45 s, followed by an extension at 72 °C for 10 min. Primers HaemF and HaemR2 were employed to detect *Plasmodium* spp. and *Haemoproteus* spp. and HaemFL and HaemR2L primers were used to detect *Leucocytozoon* spp. Many of the feather extractions yielded low DNA concentrations; thus, we established a 4 ng/µl threshold, and we tested all feather samples above that concentration, since this was the lowest DNA concentration that produced an amplicon in preliminary tests. Amplicons were visualised on a 1.5% agarose gel dyed with SYBR Safe (Thermo Fisher Scientific). Molecular grade water was used as a negative control and genomic DNA from *Plasmodium bergei* ANKA or genomic DNA from *Leucocytozoon* spp. were used as positive controls.

### Gene sequencing

Positive PCR products from all bird organs, blood and feather samples produced a ~ 470 bp amplicon, and were sequenced using the Sanger dideoxy method from the 3’ direction (Source Bioscience, Nottingham, UK) with either the primer HaemF, or HaemFL^32^, except for sample replicates from section "Feather DNA yield and PCR performance". Additionally, some randomly selected feather derived amplicons from section "Gene sequences match between samples" and representative sequences from section "Leucocytozoon spp. detection from feathers of a captive bird population" were sequenced in both directions and edited in Bioedit^33^. All sequences obtained were compared to avian haemosporidian sequences previously published in the GenBank nucleotide database using BLASTn to identify the genus of the parasite. Unique sequences found in this study were deposited in GenBank (accession numbers: OQ435376, OQ418139 and OQ418138).

## Ethics approval

All the work included in this study involving living organisms was done following international ethical procedures which fall within the ARRIVE guidelines (https://arriveguidelines.org) and following the best veterinary practices and regulations for animal handling, sample collection and euthanasia (i.e., American Veterinary Medical Association​, Guidelines for the Euthanasia of Animals (2020)). This work was done by staff with comprehensive bioethics understanding (i.e., veterinarians) and was approved by the Chester Zoo Science Committee and the University of Liverpool Veterinary Research Ethics Committee (reference VREC532a).

## Detection of haemosporidian DNA from feathers

This section aims to assess the plausibility of parasite DNA detection by examining feathers of carcasses of susceptible bird species with confirmed haemosporidian infection by organ testing. Corvids, which are prone to haemosporidian infections^34^, and other wild birds, were collected. We obtained carcasses of corvids that were culled from March to May of 2018 and 2019 as part of an annual population management program in Cheshire, United Kingdom (UK). Carcasses remained in the field for up to one week. Free-living wild birds that were found dead on the premises of Flamingo Land (Kirby Misperton, Malton, UK) and Chester Zoo (Upton, Chester, UK) during 2017 and 2018 were also sampled. In this case, carcasses remained in the field for up to three days. Feathers were collected prior to PME and stored at − 20 °C. Brain and liver samples were taken at PME, and DNA was extracted and tested for haemosporidian DNA by PCR. Feathers from birds with organs that tested positive for haemosporidian DNA were subsequently tested by PCR.

From 43 corvids received, 15 (34.8%) were positive for haemosporidian DNA in organ samples (Eurasian magpies (*Pica pica*) 13/28, Carrion crows (*Corvus corone*) 2/14 and Eurasian jackdaw (*Corvus monedula*) 0/1). Sufficient DNA for PCR testing was recovered from RF of all organ-positive birds (n = 15) and CF of only one bird. The concentration of DNA from RF ranged from 6.4 to 352 ng/µl (median = 17.7 ng/µl). The highest value was obtained from a feather that contained dry blood in the calamus. Haemosporidian DNA in feathers was detected by PCR in 11 (73.3%) of the 15 organ-positive birds. *Plasmodium* spp., *Leucocytozoon* spp. and *Haemoproteus* spp. DNA was detected in feathers of 100% (1/1), 75% (9/12) and 50% (2/4) of organ-positive birds, respectively (Table 2).

**Table 2:** Feather DNA concentration and PCR results from confirmed haemosporidian infected dead wild corvids in 2018 and 2019.

Bird ID	Species	Body weight (g)	Organ PCR		Covert feathers		Remiges and rectrices	
			Br	Li	DNA (ng/µl)	PCR result	DNA (ng/µl)	PCR result
C10	Eurasian magpie (*Pica pica*)	150	(−)	L	< 4^♦^	nt	14.4	(−)
C37	Eurasian magpie (*Pica pica*)	151	L	L	< 4^♦^	nt	74.4	(+) L
C13	Eurasian magpie (*Pica pica*)	156	H	H	< 4^♦^	nt	24.9	(+) H
C6	Eurasian magpie (*Pica pica*)	164	(−)	L	< 4^♦^	nt	17.1	(−)
C12	Eurasian magpie (*Pica pica*)	172	(−)	H, L	< 4^♦^	nt	32.9	(+) H, L
C14	Eurasian magpie (*Pica pica*)	176	H	H, L	< 4^♦^	nt	9.5	(−)
C35	Eurasian magpie (*Pica pica*)	177	L	L	< 4^♦^	nt	12.1	(+) L
C33	Eurasian magpie (*Pica pica*)	180	L	L	< 4^♦^	nt	8.7	(+) L
C31	Eurasian magpie (*Pica pica*)	186	L	L	< 4^♦^	nt	41.5	(+) L
C5	Eurasian magpie (*Pica pica*)	187	(−)	L	< 4^♦^	nt	6.4	(+) L
C20	Eurasian magpie (*Pica pica*)	188	L	L	5.1	(+)	21.3	(+) L
C11	Eurasian magpie (*Pica pica*)	190	H	H	< 4^♦^	nt	52.8	(−)
C34	Eurasian magpie (*Pica pica*)	195	L	L	< 4^♦^	nt	8.3	(+) L
C42	Carrion crow (*Corvus corone*)	427	(−)	L	< 4^♦^	nt	15.6	(+) L
C45	Carrion crow (*Corvus corone*)	462	P	P	< 4^♦^	nt	352*	(+) P

Br: Brain; Li: Liver; P: *Plasmodium* spp.; H: *Haemoproteus* spp.; L: *Leucocytozoon* spp.; (−): negative PCR result; (+): positive PCR result; nt: Not tested.

^♦^DNA concentration was too low for PCR testing.

*The feather calamus contained abundant dried blood.

From the dead wild birds collected in zoos, haemosporidian DNA was detected in 30 (23.3%) out of 129 birds using organ samples. Sufficient DNA for PCR testing was obtained from RF of 28 and CF of 21 of the 30 organ-positive birds. No feather DNA was recovered from the two smallest birds, Goldcrest (*Regulus regulus*) and Firecrest (*Regulus ignicapilla*). Overall, haemosporidian DNA was detected in feathers from 22 of the 28 birds that yielded feather DNA (78.6%). Haemosporidian DNA was detected in 19 of 28 RF (67.9%) and in 16 of 21 CF (76.2%). Detection of haemosporidian DNA in feathers was successful in 85.7% (12/14), 83.3% (5/6) and 70% (7/10) of the birds, which were organ-positive for *Leucocytozoon* spp., *Plasmodium* spp. and *Haemoproteus* spp., respectively (Table 3).

**Table 3:** Feather DNA concentration and haemosporidian PCR results from organ-positive dead free-living wild birds found in Flamingo Land and Chester Zoo during 2017 and 2018.

Bird ID	Species	Body weight (g)	Year	Organ PCR		Covert feathers		Remiges and rectrices	
				Br	Li	DNA (ng/µl)	PCR result	DNA (ng/µl)	PCR result
M37	Firecrest (*Regulus ignicapilla*)	3	2017	H^a^	H	< 4^♦^	nt	< 4^♦^	nt
M35	Goldcrest (*Regulus regulus*)	5	2017	H^b^	H	< 4^♦^	nt	< 4^♦^	nt
Me2	Northern wren (*Troglodytes troglodytes*)	6	2018	H	H	6.4	(+) H	5.6	(−)
MF3	Common swift (*Apus apus*)	13	2017	H^a^	H	< 4^♦^	nt	7.9	(−)
M103	Willow warbler (*Phylloscopus trochilus*)	15	2017	H^a^	H	< 4^♦^	nt	12.4	(+) H
MF5	Coal tit (*Periparus ater*)	16	2017	H	H	21.2	(+) H	15.5	(+) H
M25	Coal tit (*Periparus ater*)	16	2017	L	L	< 4^♦^	nt	61.9	(+) L
M38	European greenfinch (*Chloris chloris*)	20	2017	L^b^	L	< 4^♦^	nt	17.6	(−)
MF4	Song thrush (*Turdus philomelos*)	79	2017	H	H	< 4^♦^	nt	18.7	(−)
Me3	Eurasian blackbird (*Turdus merula*)	82	2018	P	P	7.6	(−)	12.6	(−)
M92	Eurasian blackbird (*Turdus merula*)	85	2017	H	(−)	< 4^♦^	nt	18.5	(+) H
M89	Eurasian blackbird (*Turdus merula*)	88	2017	H	L	8.2	(+) L	28.6	(+) H, L
Me4	Eurasian blackbird (*Turdus merula*)	88	2018	P	P	5.1	(+) P	12.9	(+) P
M26	Eurasian blackbird (*Turdus merula*)	90	2017	P	P	29.3	(+) P	19.7	(+) P
M93	Eurasian blackbird (*Turdus merula*)	90	2017	H	H	33.9	(+) H	37.9	(+) H
M112	Eurasian blackbird (*Turdus merula*)	95	2017	P	P	24.2	(+) P	13.2	(+) P
Me5	Eurasian blackbird (*Turdus merula*)	95	2018	P	P	9.3	(+) P	33.3	(−)
Me1	Eurasian blackbird (*Turdus merula*)	96	2018	P	P	< 4^♦^	nt	15.1	(+) P
M90	Eurasian blackbird (*Turdus merula*)	100	2017	H	L	16.3	(+) H, L	10.6	(+) L
M113	Eurasian blackbird (*Turdus merula*)	100	2017	(−)	L	63.9	(+) L	27	(+) L
M125	Eurasian magpie (*Pica pica*)	170	2017	L	L	8.7	(−)	22.7	(+) L
Me7	Eurasian jackdaw (*Corvus monedula*)	170	2018	(−)	L	23.7	(−)	12.2	(+) L
M84	Carrion crow (*Corvus corone*)	180	2017	L	L	5.7	(+) L	7.1	(+) L
M101	Common moorhen (*Gallinula chloropus*)	280	2017	L^b^	(−)	11	(+) L	9.3	(−)
MF9	Rock dove (*Columba livia*)	420	2017	L	L	59.5	(+) L	29.2	(+) L
M111	Rock dove (*Columba livia*)	450	2017	L	L	8.2	(+) L	12	(+) L
M127	Rock dove (*Columba livia*)	470	2017	L	L	50.4	(+) L	14.4	(+) L
MF10	Tawny owl (*Strix aluco*)	480	2017	L	L	6.9	(+) L	25.6	(+) L
M94	Eurasian buzzard (*Buteo buteo*)	790	2017	(−)	H	14.5	(−)	9.1	(−)
M7	Mallard (*Anas platyrhynchos*)	1000	2017	L^b^	L	7.6	(−)	11.4	(−)

Br: Brain; Li: Liver; P: *Plasmodium* spp.; H: *Haemoproteus* spp.; L: *Leucocytozoon* spp.; (−): negative PCR result; (+): positive PCR result; nt: Not tested.

^a^First report of haemosporidians in this bird species.

^b^First report of the parasite genus in this bird species.

^♦^DNA concentration was too low for PCR testing.

## Environment effect on feather DNA yield and PCR results

Carcasses and shed feathers in the wild are exposed to DNA-degrading conditions, like humidity, rain, UV radiation and temperature changes. Here, we evaluated environmental effects on feather DNA extraction and subsequently, on parasite detection, to identify optimum storage conditions. A single specimen was used to eliminate the variation among individuals observed in section "Detection of haemosporidian DNA from feathers". Feathers were obtained in 2018 from Chester Zoo from a Red-breasted goose (*Branta ruficollis*) naturally infected with *Plasmodium* spp. The infection was diagnosed from blood samples by PCR and observing blood smears when the bird was hospitalised. It presented a high intensity of infection (4.5%)^35,36^ and was euthanised due to poor prognosis; organ samples were also positive for *Plasmodium* DNA by PCR. A total of 16 remiges were collected shortly after death and immediately stored in a plastic bag at − 20 °C. After two months, two feathers were taken for DNA extraction and quantification to use as a control; the experimental feathers were separated into three groups with different storage conditions: outdoors, in a room and inside a freezer. In September, at the University of Liverpool, two feathers were placed in a punctured plastic bag in the outdoors location, six feathers were placed in a punctured plastic bag and left in a room without temperature control, exposed to artificial and natural light, and the other six feathers remained frozen in a plastic bag at − 20 °C. After two months (November), two feathers from each location were taken for DNA extraction; while after four (January) and six months (March), two feathers from the room and freezer locations were taken for DNA extraction. Extraction was done individually for each feather using the content of the rachis; all extractions were quantified by spectrophotometry and subsequently tested by PCR. Temperature and relative humidity (RH) in the outdoor and room locations were recorded with Tinytag Ultra2 (Gemini) loggers, programmed to record every hour. The average temperature in the outdoors location for the experiment period (26th of September 2018 to 26th of November 2018) was 9.5 °C (min − 3.1 °C, max 26.6 °C), and the average RH was 89.1% (min 39.7%, max 100%); for the indoors location, they were 24 °C (min 8.5 °C, max 32.2 °C) and 52.4% (min 32.9%, max 82.2%), respectively. DNA concentration from the experimental feathers was compared to the average of the control with a t-test for one sample and the data was log_10_ transformed. A Mann–Whitney-U test was used to compare the amount of DNA obtained at room temperature and freezer. Statistical analyses were done using R Studio™^37^. The number of observations from the feathers kept outside was insufficient for analysis.

The average DNA concentration of feathers that remained in the freezer for two (264.2 ng/µl), four (294.8 ng/µl) and six (242.5 ng/µl) months was similar to that of control feathers (273 ng/µl), and these values did not vary significantly among each other (t-test, t = − 0.18, *P* = 0.86). In contrast, there was a significant reduction in the average DNA concentration from feathers kept at room temperature for two (47.6 ng/µl), four (59.4 ng/µl) and six (45.9 ng/µl) months; when compared to the control (t-test, t = − 36.52, *P* < 0.001), or with frozen feathers (Mann Whitney U-test, W = 36, *P* = 0.002). Storage at room temperature and in the freezer showed little variation in DNA concentration over time. For the feathers kept outdoors, the average DNA concentration retrieved was 22.8 ng/µl. *Plasmodium* spp. was detected from the control feathers, as well as those kept in the freezer and at room temperature at all time points despite the significant decrease in DNA concentration in the latter case, but it was not detected in feathers left outdoors (Table 4).

**Table 4:** Feather DNA extraction and PCR results from feathers kept at different temperature conditions for 2, 4 and 6 months.

Storage time	0 months^a^		2 months		4 months		6 months	
	DNA (ng/µl)	PCR result	DNA (ng/µl)	PCR result	DNA (ng/µl)	PCR result	DNA (ng/µl)	PCR result
Freezer	230 .2 315.8 (273)	(+) (+)	324.6 203.8 (264.2)	(+) (+)	336.4 253.1 (294.8)	(+) (+)	147.2 337.7 (242.5)	(+) (+)
Room temperature			66.8 28.4 (47.6)	(+) (+)	53.9 64.9 (59.4)	(+) (+)	52.7 39.0 (45.9)	(+) (+)
Outdoors			16.4 29.1 (22.8)	(−) (−)				

Two replicates were used per treatment and DNA concentration averages are shown in parentheses.

^a^DNA extractions used as a positive control.

## Feather DNA yield and PCR performance

The aim of this section was to assess variation in DNA yield across different bird species and feather types, and how that yield affected the detection of haemosporidian DNA by PCR. Consequently, this allowed us to determine the number and type of feathers required for haemosporidian PCR detection. Feathers for this section derived from carcasses that were frozen immediately after death to remove the environmental effect on feather DNA yield observed in previous sections. For each bird, a subset of different feather types and numbers were collected, each with five repeats, so that individual variation was removed, and statistical power increased. The Gower Bird Hospital (GBH) (Swansea, Wales, UK) provided carcasses of rescued birds that died under their care or that, due to poor prognosis, were euthanized (n = 8). A blood sample per bird was taken shortly after death; samples and carcasses were immediately stored at − 20 °C. Prior to PME, feathers were collected and stored at − 20 °C. DNA was extracted from organs and blood samples and tested for haemosporidian DNA by PCR. DNA was extracted by pooling feathers with five repeats per group as follows, CF: 1, 5, 10, 15 and 20, RF: 1, 2 and 3, for a total of 320 feather pools. Feather DNA extracts of bird W6 were not tested by PCR due to laboratory access restrictions during the Covid19 pandemic.

Regression analysis explored the factors influencing DNA yield and PCR outcome separately. The relationship between feather type and the number of feathers per pool was analysed with a linear mixed model where the DNA concentration was the dependent variable, the feather group (CF1, CF5, CF10, CF15, CF20, RF1, RF2, and RF3) was a fixed effect and bird was a random effect. The effect of body weight on DNA yield was analysed with a linear model. The PCR outcome (positive or negative) was analysed with a generalised linear model using a binomial family and DNA concentration, weight, and feather group as explanatory variables. Bird W6 was excluded from the latter analysis as PCR was not undertaken on its samples, but the DNA concentration derived from its feathers was used in the analyses of factors affecting the DNA yield. The DNA concentration (ng/µl) was log_10_ (n + 1) transformed to achieve normality. Best models were selected by the lowest Akaike information criterion and model’s assumptions were confirmed with histograms of residuals, Q–Q plots and plots of fitted values versus residuals. Percentiles were estimated for positive (5th) and negative (95th) PCR results in relation to the most significant variable, and the simplest adequate model was used to produce probability predictions. All statistical analyses were done using R Studio™, package *lme4*^37^.

Testing of blood samples by PCR detected two birds infected with *Haemoproteus* spp. and five infected with *Leucocytozoon* spp. Testing of brain samples by PCR uncovered four *Leucocytozoon* spp. infections, three mixed infections of *Haemoproteus* spp. and *Leucocytozoon* spp., and one mixed infection of *Plasmodium* spp. and *Leucocytozoon* spp. (Supplementary Table S1).

**Figure 1:**
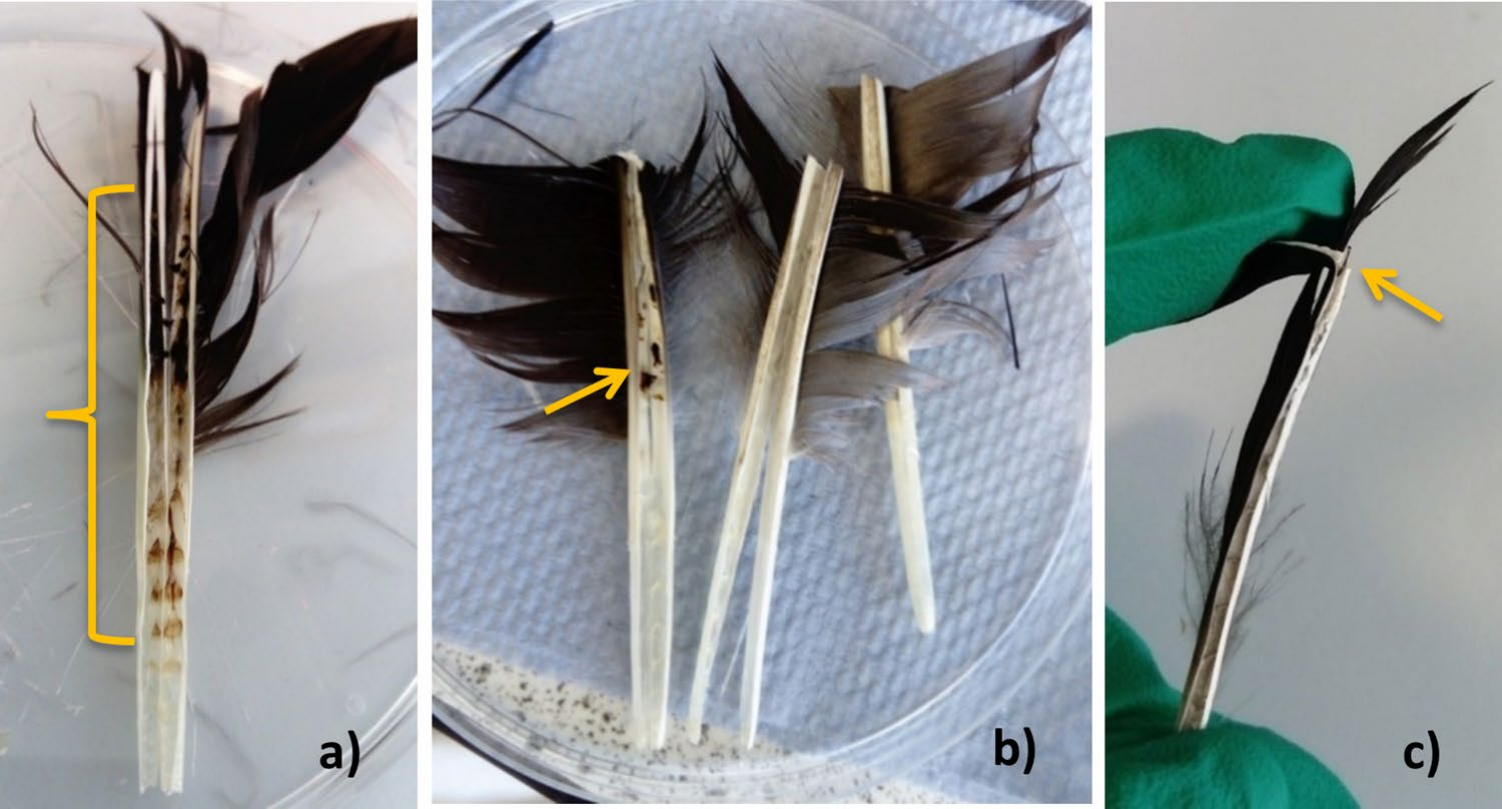
Feathers with dry blood inside the shaft. (**a)** The blood is located from the superior umbilicus to the distal portion of the rachis; (**b)** the blood is in a small section of the rachis, from the superior umbilicus to the distal part of the rachis; (**c)** the blood is only observable at the distal portion of the rachis. Bracket and arrows indicate blood’s location.

DNA was extracted from all CF and RF groups, except for the group CF1 from bird W2 (Eurasian jackdaw). DNA yield ranges observed were as follows: CF1 (0.4–26.5 ng/µl), CF5 (2–52.5 ng/µl), CF10 (2.3–153.2 ng/µl), CF15 (2.8–199.2 ng/µl), CF20 (5.7–188.4 ng/µl), RF1 (10.3–2131.1 ng/µl), RF2 (11.9 to 584.5 ng/µl) and RF3 (23.5–623.9 ng/µl) (Table 5). On some occasions, dry blood was observed inside the feathers’ shaft distributed in different sections of the rachis (Fig. 1); DNA extractions of these feathers generally produced higher DNA yields than that observed for other groups of the same kind and number of feathers.

**Table 5:** DNA concentration after extraction of covert feathers, remiges and rectrices pooled into different numbers with five replicates by feather group.

Bird ID (g)	CF1 (ng/µl)	CF5 (ng/µl)	CF10 (ng/µl)	CF15 (ng/µl)	CF20 (ng/µl)	RF1 (ng/µl)	RF2 (ng/µl)	RF3 (ng/µl)
W6^♦^ Eurasian blackbird (59)	2.5	4.5	6	8.2	9.8	12.1	29.6	40.8
	3.1	5.5	7.3	9.8	12.2	18.1	32.3	43.1
	3.7	6	8.1	10.6	15.8	23.4	37.5	55.3
	4	6.2	9	10.7	18.3	27.3	37.9	**-**
	5.5	7	10.4	18.1	25.2	29.4	41.5	**-**
	(3.8)	(5.8)	(8.2)	(11.5)	(16.3)	(22.1)	(35.8)	(46.4)
	± 1.1	± 0.9	± 1.7	± 3.8	± 6.0	± 7.0	± 4.8	± 7.8
W2 Eurasian jackdaw (224)	0	2	2.3	**2.8**	3	17.8	**33.3**	**66.1**^**b**^
	0.3	2.1	2.5	**3.6**	5.6	21.1	35.2	68.60
	0.4	2.3	2.6	4.3	7.6	25.2	48.9	77.9
	0.6	4.8	**3.5**	5	**12.4**	28.5	58.9	**112.5**
	1.6	5.5	4.2	**7.4**	12.9	**2131.1**^**b**^	62.3	**155.1**^**b**^
	(0.6)	(3.3)	(3.0)	(4.6)	(8.3)	(444.7)	(47.7)	(96.0)
	± 0.6	± 1.7	± 0.8	± 1.8	± 4.3	± 942.7	± 13.3	± 37.9
W1 Carrion crow (425)	3.6	6.2	10.1	11.2	**19.5**	27.7	53	**83.4**
	3.8	8.3	12.3	12.6	20.2	33	66.5	**102.6**
	4.8	**8.3**	12.6	16.5	23.6	57.7	**69.7**^**b**^	**144.7**
	8.4	8.8	**12.6**	17.7	24.2	68.4	**74.4**^**b**^	**205.8**
	9	10.1	19.6	**18.1**	**24.3**	**137.4**	**111.7**	**231.80**
	(5.9)	(8.3)	(13.4)	(15.2)	(22.4)	(64.8)	(75.1)	(153.7)
	± 2.6	± 1.4	± 3.6	± 3.1	± 2.3	± 43.9	± 22.0	± 64.1
W7 Mistle thrush (106)	4	5.2	5.1	9.3	**14.2**	**34.3**	**39.8**	**106.4**
	4.3	5.7	5.6	**10.4**	**16.9**	40.3	**76.4**	**115.9**
	5.1	**6.2**	9.3	**12.2**	**19**	**40.8**	88.3	**118.7**
	5.4	**9.4**	**9.9**	**15.7**	**19**	53.8	**100.5**	**147.6**
	6.4	9.6	12.6	**16.4**	19.8	**64.9**	**136**	190.8
	(5.0)	(7.2)	(8.5)	(12.8)	(17.8)	(46.8)	(88.2)	(135.9)
	± 1.0	± 2.1	± 3.1	± 3.2	± 2.3	± 12.4	± 35.1	± 34.3
W8 Eurasian magpie (172)	2.9	4.1	6.7	12.7	**20.3**	46.2	**79.4**	**53.2**
	2.9	**4.8**	11.3	**14.3**	**20.4**	**46.4**	**80.2**	**62.7**
	3	5.2	**12.1**	**19.4**	**21**	**70.3**	**99.2**	**79.5**
	4.4	**6**	**13**	**22.8**	**21.9**	**79.6**	**102.3**	**135.3**
	4.5	7.6	16.8	**24.7**	**28.8**	**125.7**	**123.8**	**218.7**
	(3.5)	(5.5)	(12.0)	(18.8)	(22.5)	(73.6)	(97.0)	(109.9)
	± 0.8	± 1.3	± 3.6	± 5.2	± 3.6	± 32.6	± 18.3	± 68.6
W4 Song thrush (69)	2.5	2.8	**3.5**	**4.9**	**5.7**	**10.3**	11.9	23.5
	3.5	**2.8**	**5**	**5.3**	**6.9**	11	**16.8**	**28.8**
	3.6	**3**	**5.7**	**5.9**	7.2	**11.4**	**17.5**	37
	4.3	**4.4**	**5.9**	**6**	**17**	15.5	20	**42.8**
	6.1	**7**	7	**6.8**	18.1	**17.5**	**25.6**	**48.9**
	(4.0)	(4.0)	(5.4)	(5.8)	(11.0)	(13.1)	(18.4)	(36.2)
	± 1.3	± 1.8	± 1.3	± 0.7	± 6.0	± 3.2	± 5.0	± 10.3
W5 Song thrush (45)	1.6	**4**	**7.1**	**7.9**	**11.6**	**87.4**	**127.3**	**158.5**
	1.7	5.6	**8.4**	**8.2**	**11.9**	**89.6**	**158**	**221.5**
	1.7	**6.8**	9.3	**8.7**	**13.1**	**138.5**	**166.8**	**307.5**
	**2.9**	7.6	**10.1**	**10.6**	**43**	**148.4**	**308.4**	**623.9**
	3.1	**8.1**	**10.3**	**20**	**71.1**	**171.2**^**b**^	**584.5**	**725.6**
	(2.2)	(6.4)	(9.0)	(11.1)	(30.1)	(127.0)	(269.0)	(407.4)
	± 0.7	± 1.6	± 1.3	± 5.1	± 26.5	± 37.1	± 189.7	± 252.3
W3 Common raven (953)	**6.8**	**47.4**	**71.1**	**107.3**	**118.8**	**105.9**^**b**^	**103.4**^**b**^	**147**^**b**^
	**12**	**48.3**	**89.5**	**109.5**	**125**	**169.6**^**b**^	**155.8**^**b**^	**185.30**^**b**^
	**12.6**	**48.9**	**102.9**	**116.4**	**129.8**	**726.9**^**b**^	**160.7**^**b**^	**195.9**
	**22.3**	**51**	**132.9**	**177.1**	**187.4**	**726.9**^**b**^	**248**^**b**^	**227.8**^**b**^
	**26.5**	**52.2**	**153.2**	**199.2**	**188.4**	**868.4**^**b**^	**386**^**b**^	**524.4**^**b**^
	(16.0)	(49.6)	(109.9)	(141.9)	(149.9)	(519.5)	(210.8)	(256.1)
	± 8.1	± 2.0	± 33.1	± 43.1	± 34.9	± 354.0	± 110.8	± 152.7

CF: Covert feathers; RF: Remiges and rectrices; DNA concentration average is given in parenthesis; ± : Standard deviation.

^b^Blood was observed in the feathers.

^♦^This bird´s DNA extracts were not tested by PCR.

**−**: DNA was not recovered after extraction. Bold Values indicate positive PCR results. Birds are organised from fewer to more PCR positive results.

**Figure 2:**
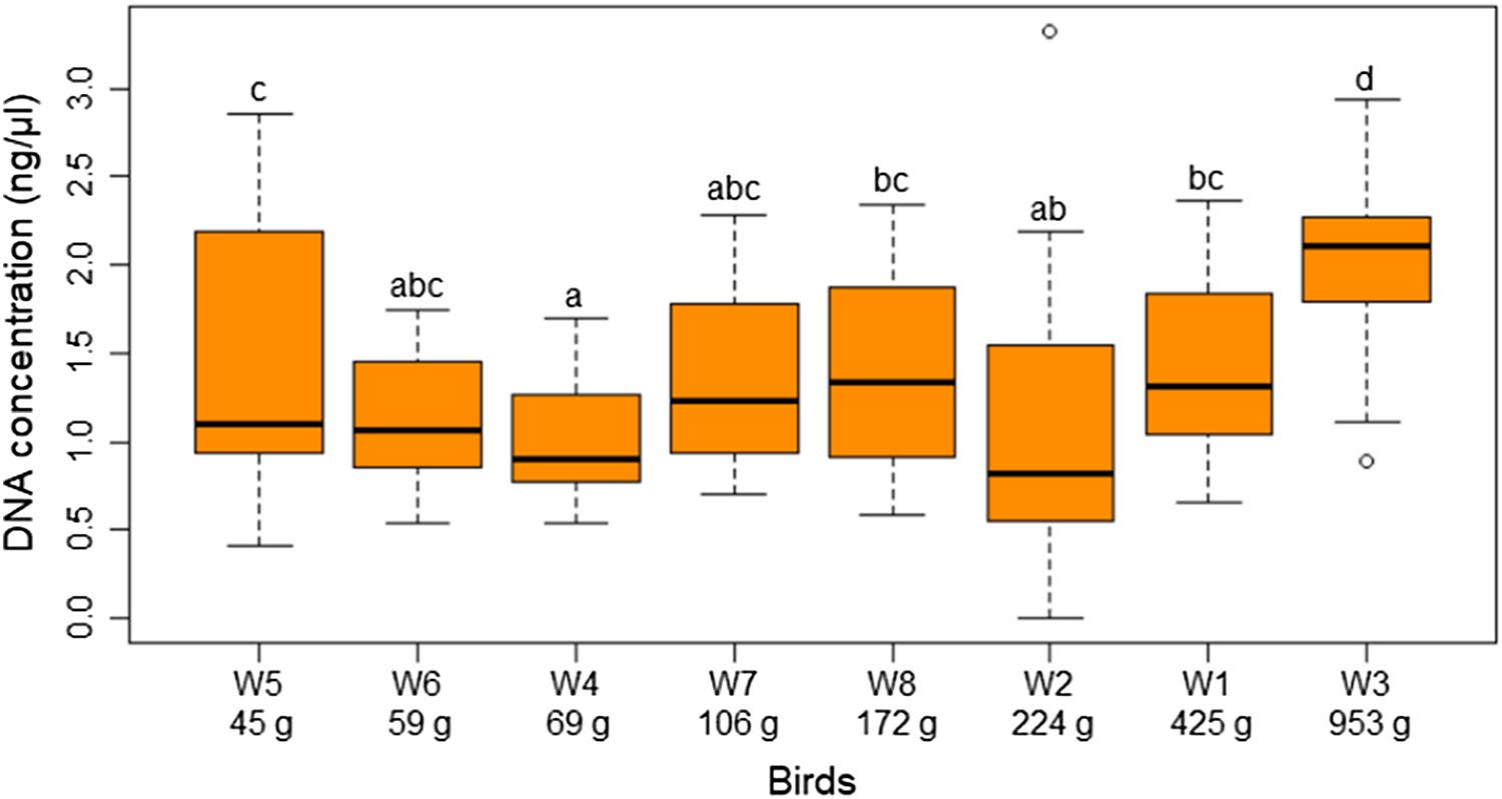
DNA concentration extracted for all feathers of each bird from Gower Bird Hospital. Data was log_10_ (n + 1) transformed prior to analysis. A least square means test with a Tukey adjustment was used to find significant differences among birds. For all boxes with the same letters the difference between the means is not statistically significant. Birds are ordered from lightest to heaviest. See Supplementary Table S1 for bird details.

**Figure 3:**
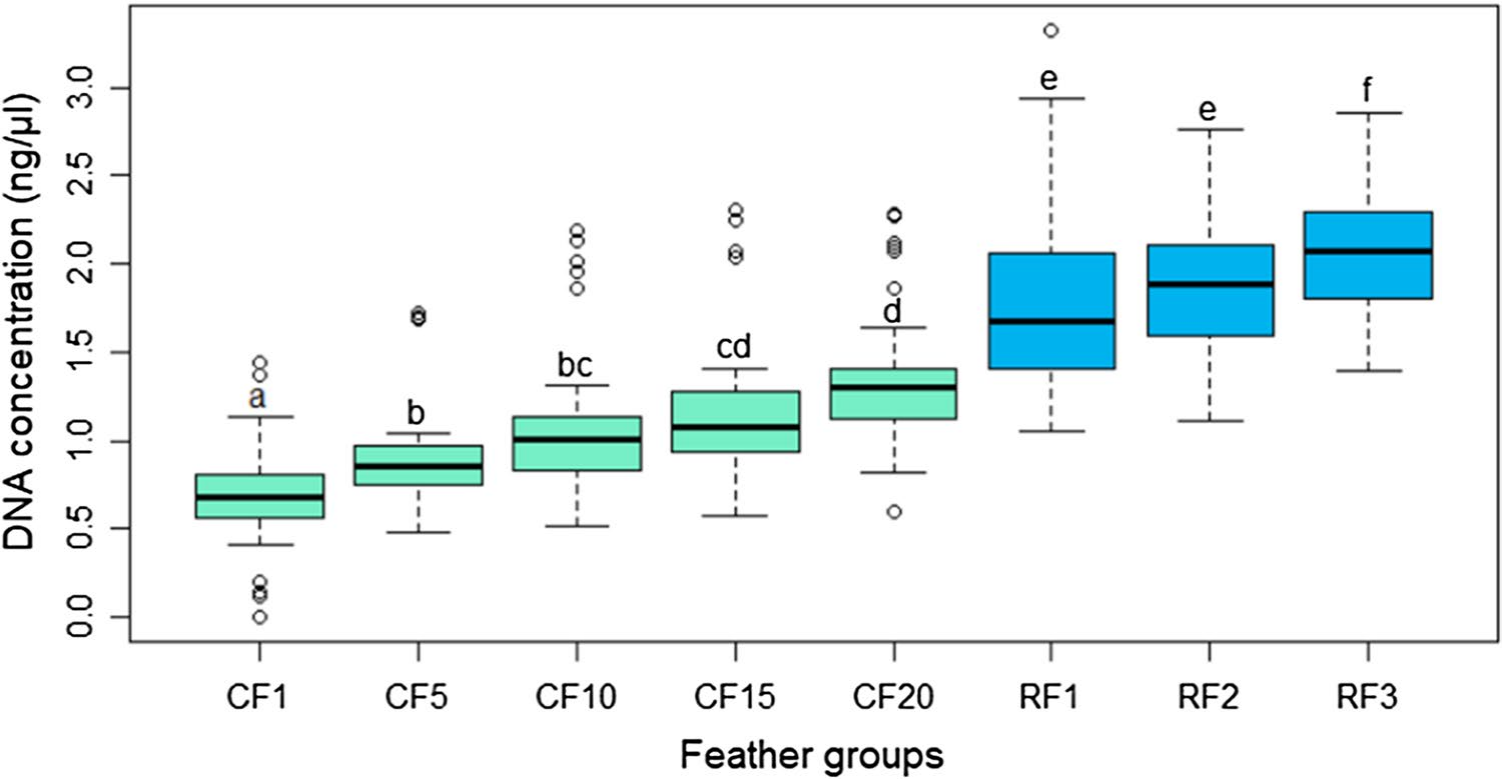
DNA concentration extracted by feather groups for all birds of the Gower Bird Hospital. A least square means test with a Tukey adjustment was used to find significant differences among feather groups. For all boxes with the same letters the difference between the means is not statistically significant. Data was log_10_ (n + 1) transformed prior to analysis.

Birds´ body weight (n = 8) influenced the amount of DNA extracted, with the heavier birds yielding more DNA (F = 70.89, df = 316, *P* < 0.001). The DNA concentration varied among birds with the Common raven (W3) being significantly different from all others (Fig. 2). The DNA concentration per feather group varied among birds and was significantly different (F = 157.6, df = 303, *P* < 0.001). It was noticed that the DNA concentration improved as the number of feathers used per pool increases and that the RF groups produced a higher DNA concentration than the CF groups (Fig. 3). The DNA concentration by feather group per bird can be found in the Supplementary information (Figure S3). Interestingly, in both the Common raven (W3) and the Eurasian jackdaw (W2), one RF yielded higher DNA concentration than two or three RF.

From the seven birds screened for haemosporidian DNA by PCR (W6 was not tested), parasites were detected in all CF and RF groups except for CF1 (2/7 birds) and CF5 (6/7 birds). Only in the Common raven (*Corvus corax*) (bird W3), haemosporidian DNA was detected in every feather group. The lowest rate of detection was in the Eurasian jackdaw (*C. monedula*) (bird W2), with only 10 positive feather groups out of 40. High standard deviation values for RF originated from high DNA concentrations from feathers with visible blood (Table 5). The DNA concentration was the variable that influenced the PCR outcome the most (Z = − 6.001, df = 2.79, *P* < 0.001).

The 5th percentile of DNA concentration in relation to PCR positive results was estimated at 4.58 ng/µl, meaning that 95% of the positive samples had a concentration higher than this value. Likewise, the 95th percentile of DNA concentration regarding negative results was found at 64.61 ng/µl, meaning that 95% of negative samples had a DNA concentration lower than this value. The simplest adequate model including only DNA extraction as an explanatory variable and PCR outcome as the dependent variable was used to predict the probability of a positive result. For the mentioned percentiles it was, 29.2% for 4.58 ng/µl and 81.8% for 64.61 ng/µl; additionally, the 50% probability of a positive result was found with 12.2 ng/µl.

### Gene sequences match between samples

To further determine the correspondence between haemosporidian DNA recovered from blood samples with that from feathers, sequence identity for both groups was compared. Positive PCR products from two flight feathers and two covert feathers per bird were randomly selected and sent for sequencing in both directions by the Sanger method with the primers HaemF and HaemR2^32^. Merged forward and reverse feather sequences of the same bird were aligned and trimmed to produce sequences of the same length to compare with the corresponding blood or brain sequence using the BioEdit© software^33^.

The genetic analysis comparing haemosporidian DNA sequences from feathers (n = 4; two CF and two RF) and blood of the same bird revealed a complete correspondence for the birds W8 and W5 and 99.7% similarity for bird W2. For the other birds, the level of agreement between blood and feather sequences ranged from 90.5 to 100%, with at least one of the feathers matching the sequence observed in the blood (Table 6). For bird W1, DNA was not extracted from blood, and so brain and feather sequences were compared instead. For birds W2 and W3, only forward feather sequences were used for comparison due to low quality of the reverse sequences.

**Table 6:** Comparison of brain or blood sequences against feather sequences from the same birds.

Bird ID	Species	Reference sequence	Genus	Sequence length^a^	Feathers group			Similarity^b^ (%)
					(n)	Type	R	
W1	Carrion crow (*Corvus corone*)	Brain	L	438	20	CF	4	100
					2	RF	1	91.7
					2	RF	3	92.5
					15	CF	3	92.5
W2	Eurasian jackdaw (*Corvus monedula*)	Blood	L	442	1	RF	1	99.7
					2	RF	4	99.7
					15	CF	5	99.7
					20	CF	3	99.7
W3	Common raven (*Corvux corax*)	Blood	L	443	1	CF	1	100
					1	CF	2	97.1
					1	RF	2	97.1
					1	RF	3	96.2
W4	Song thrush (*Turdus philomelos*)	Blood	H	431	3	RF	3	100
					20	CF	4	100
					20	CF	3	100
					3	RF	4	98.8
W5	Song thrush (*Turdus philomelos*)	Blood	L	447	1	RF	1	100
					1	RF	2	100
					20	CF	2	100
					20	CF	4	100
W7	Mistle thrush (*Turdus viscivorous*)	Blood	L	455	1	RF	1	97.8
					2	RF	5	90.7
					20	CF	2	90.7
					20	CF	4	90.5
W8	Eurasian magpie (*Pica pica*)	Blood	L	405	1	RF	2	100
					1	RF	3	100
					15	CF	2	100
					15	CF	5	100

^a^Feather sequence lengths, given in base pairs; R: identifying number of the replicate used (chosen randomly).

^b^Similarity percentage of the feather sequences compared to the reference blood or organ sequence from the same bird. L: *Leucocytozoon* spp.; H: *Haemoproteus* spp.; CF: Covert feather; RF: Remiges and rectrices; bp: base pairs; (n): number of feathers used per pool.

## Detection of haemosporidian DNA from feathers in a wild bird population

The objective of this section was to test the effectiveness of haemosporidian detection using feathers naturally shed during handling of wild birds, a common ornithological scenario. Eurasian blackbirds (*Turdus merula*) are commonly infected with haemosporidians^38^, and they have been found infected in Chester Zoo^39^. Therefore, bird-ringing groups from Merseyside were asked to collect Eurasian blackbird feathers that came loose during handling. Once collected, feathers were kept at room temperature for variable periods, up to seven months, before posting them to the University of Liverpool. Feathers from 71 birds were received, ranging from one to 23 per individual, and all were CF except for two RF. Samples came from different locations in Wales and they were taken between February and June 2018. Feather DNA was extracted by pooling from one to 23 calami per bird. Haemosporidian DNA detection by PCR testing of these samples was repeated on four occasions, increasing the DNA amount used as template and reducing the water volume proportionally to keep the final volume at 20 µl for the first amplification; each test used 1, 2, 3, and 4 µl of DNA template.

From the DNA extractions of the Eurasian blackbirds (n = 71), only eight yielded sufficient DNA for PCR testing (> 4 ng/µl) ranging from 4.5 to 13.6 ng/µl. The use of one, two and three microliters of template for the PCR produced negative results (n = 8), but a successful haemosporidian DNA detection, identified as *Plasmodium* spp. was obtained using four microliters (Table 7).

**Table 7:** Testing of Eurasian blackbird feathers by PCR for haemosporidian.

Bird ID	Sampling date	Ringing site	CF (n)	DNA (ng/µl)	PCR template used			
					1 µl	2 µl	3 µl	4 µl
BB2	21/04/2018	Weaver valley	23	13.6	(−)	(−)	(−)	(−)
BB19	11/03/2018	Woodford	5	13.5	(−)	(−)	(−)	(−)
BB10	05/07/2017	Bromborough	23	11.4	(−)	(−)	(−)	(+)
BB14	11/03/2018	Woodford	8	8.1	(−)	(−)	(−)	(−)
BB24	10/03/2018	Burton	3	6.5	(−)	(−)	(−)	(−)
BB65	04/03/2018	Houghton	4^♦^	5.8	(−)	(−)	(−)	(−)
BB20	11/03/2018	Woodford	3	5.7	(−)	(−)	(−)	(−)
BB21	11/03/2018	Woodford	3	4.5	(−)	(−)	(−)	(−)

*CF* covert feathers.

^♦^Two remiges were received from this bird and used for DNA extraction.

## *Leucocytozoon* spp. detection from feathers of a captive bird population

A final test was done to measure the effectiveness of the method for detecting haemosporidian infections in live wild birds of a captive population with unknown haemosporidian prevalence. A Sumatran laughingthrush (SL) (*Garrulax bicolor*) population from Chester Zoo was chosen because *Haemoproteus* spp. and *Leucocytozoon* spp. were detected in SL of other aviaries on previous screening. Zoo staff collected feathers that came loose during routine handling of the birds (n = 22) and recently moulted feathers from the bird enclosure (n = 30). Identity of the birds was recorded when the feathers were obtained from handling; however, when the feathers were collected from the enclosure, the birds’ identity was assumed based on the proximity of the feathers to the corresponding nesting site. Feathers were stored in plastic bags at − 20 °C.

Two or three RF or up to 20 CF were pooled for each DNA extraction but smaller pools were made when not enough feathers were available. The calculation was based on the amount of DNA recovered from feathers of birds with a similar weight to the SL in a previous experiment (section "Feather DNA yield and PCR performance") and a possible decrease in yield from exposure to the environment. For DNA extraction of RF, the basal rachises were cut into small pieces (Supplementary Figure S1), and the optional step of centrifuging and transferring the supernatant to a 1.5 ml reaction tube after the incubation was carried out. For the PCR, the DNA template volume was increased from 1 to 5 µl, and the water volume was adjusted accordingly.

Feather DNA extractions from 11 different SL yielded concentrations from 2.8 to 46 ng/µl employing between two and 10 CF, and 4.8 to 337.9 ng/µl when two to three RF were used. Results are reported for a group of birds when feathers could not be associated with individuals. Haemosporidian DNA was detected by PCR in four individuals (C17703, C14419, C18421 and C12640) and in three groups on two occasions each (C15751/C15752/C181038, C14418/C16484 and C18663/C18857) (Table 8).

**Table 8:** *Leucocytozoon* spp. detection from Sumatran laughingthrushes feathers from Chester Zoo.

Bird ID	Sampling date	CF (n)	RF (n)	DNA (ng/µl)	PCR result
C15751/C15752/C181038	NR	9		2.8	(−)
C18421	NR	2		3	(−)
C15752	02/11/18	10		3.6	(−)
C17703	16/10/18	2		4.7	(+)
C14419/C16484	01/01/19		2–3	4.8	(−)
C18683	NR		2–3	5.2	(−)
C15751/C15752/C181038	01/01/19		2–3	6.9	(+)
C14419/C16484	01/01/19		2–3	8.6	(+)
C15751	02/11/18		2–3	8.9	(−)
C15751/C15752/C181038	NR		2–3	9.6	(−)
C15749/C15750	16/10/18		2–3	9.8	(−)
C15751/C15752/C181038	01/01/19		2–3	9.9	(−)
C14419	20/11/18		2–3	10.7	(+)
C15749	17/10/18	10		10.7	(−)
C181038	02/11/18	2		10.7	(−)
C15751/C15752/C181038	01/01/19		2–3	11.2	(−)
C15751/C15752/C181038	01/01/19		2–3	11.3	(+)
C18683	NR	6		12.7	(−)
C18421	21/11/18		2–3	13.1	(+)
C18663/C18857	01/01/19		2–3	13.5	(+)
C15751/C15752/C181038	01/01/19		2–3	14	(−)
C18538	NR	2		15.3	(−)
C15751/C15752/C181038	01/01/19		2–3	17	(−)
C15751/C15752/C181038	01/01/19		2–3	17.2	(−)
C15749/C15750	16/10/18		2–3	17.3	(−)
C15751/C15752/C181038	01/01/19		2–3	18.9	(−)
C15751/C15752/C181038	01/01/19		2–3	19.3	(−)
C18421	02/01/19		2–3	19.7	(−)
C14419	20/11/18		2–3	20.2	(−)
C14419/C16484	01/01/19		2–3	22	(+)
C15751/C15752/C181038	NR		2–3	22.1	(−)
C18663/C18857	01/01/19		2–3	22.2	(−)
C15751/C15752/C181038	01/01/19		2–3	22.4	(−)
C18421	21/11/18		2–3	22.5	(−)
C18663/C18857	01/01/19		2–3	23.7	(−)
C15751/C15752/C181038	01/01/19		2–3	23.9	(−)
C18421	02/01/19		2–3	24.2	(−)
C14419/C16484	01/01/19		2–3	25.1	(−)
C18421	21/11/18		2–3	26	(−)
C18663/C18857	01/01/19		2–3	27.6	(−)
C18857	NR	8		28.1	(−)
C18663/C18857	01/01/19		2–3	29.6	(−)
C18857/C18683	21/11/18		2–3	32.5	(−)
C18663/C18857	01/01/19		2–3	33.3	(−)
C18663/C18857	01/01/19		2–3	38.6	(+)
C15751/C15752/C181038	01/01/19		2–3	40.1	(−)
C15750	17/10/18	3		40.3	(−)
C18857/C18683	21/11/18		2–3	42.5	(−)
C15751	02/11/18	8		46	(−)
C18421	02/01/19		2–3	59.4	(+)
C16484	20/11/18	7		63.2	(−)
C12640	06/11/18		2–3	337.9	(+)

CF: Covert feather; RF: Remiges and rectrices; (n): number of feathers used; NR: Not recorded.

## Discussion

This work shows that haemosporidian parasite DNA can be detected from feathers as an alternative to blood or organ samples and that there are relevant factors that affect the detection. DNA concentration was critical for the sensitivity of the PCR test, and it was influenced by exposure to environmental conditions, sample preservation, bird weight, feather type, number of pooled feathers and feather intrinsic variation. Overall, extracting sufficient DNA for blood parasite detection by PCR was possible for most feathers with some exceptions likely related to the state of preservation of the sample, especially in cases where the feathers were plucked from dead birds that remained in the field for an unknown time and for feathers stored at room temperature for prolonged periods.

### Feather DNA yield

The DNA yield from a single RF from different species (10.3–2131.1 ng/µl), kept at optimal conditions (frozen at − 20 °C immediately after collection), was the same or higher than what has been obtained from blood samples (30–50 ng/µl) and organs (100–800 ng/µl). Similarly, other studies have retrieved higher DNA yields from feathers (53.4 ng/µl) than from skin samples (26.7 ng/µl) of museum specimens^21^. The opposite has also been observed with lower DNA yields obtained from feathers compared to blood or tissue samples^23^; however, in that case, feathers remained in the field for a long time and were stored for 1–20 months at room temperature. It has been suggested that inappropriate preservation methods^40^ and long presence of feather in the field could result in high DNA degradation^21,23^. In addition, a direct comparison of extraction from feathers and blood samples showed that extracts of blood samples contained significantly more DNA (30.9 ± 18.8 ng/µl) than the feathers (1.2 ± 0.7 ng/µl)^19^. Differences in DNA yield may be attributed to the storage conditions and the bird’s weight from which samples were obtained. Feathers obtained from the Gower Bird Hospital (GBH) belonged to species weighing between 45 and 953 g and were immediately stored at − 20 °C, whereas feathers collected by Harvey^24^ were obtained from 11 g bird species and were stored at room temperature. We observed a marked DNA retrieval decrease when samples are stored at room temperature; likewise, we found a significant difference on DNA concentration caused by body weight, with the heaviest birds producing higher DNA amounts.

DNA concentration of feathers kept at − 20 °C was maintained relatively unaltered throughout the six months that they were stored, making this the best method to preserve feathers. Feathers kept outdoors for two months had a 91.6% decrease in DNA yield compared to feathers kept at − 20 °C. Environmental effects on feather DNA retrieval have been noted before, as Taberlet et al.^41^ state that feathers should be collected as soon as they are moulted, for if they stay long in the field, DNA will degrade. Likewise, an 86.2% decrease in DNA yield was observed in feathers stored at room temperature (24 °C) from two months onwards, compared to those kept at − 20 °C. DNA degradation is a continuous process, and it is likely that it begun before our starting experimental point of two months; afterwards, it remained stable over the experimental period until the end point of six months. It is possible that DNA concentration will not decrease further to a certain point after storage at room temperature for prolonged periods; for instance, Horváth et al.^21^ successfully extracted DNA from feathers of museum specimens over one hundred years old.

Another methodological aspect directly linked to DNA retrieval is the means of feather collection. Feathers can be obtained by plucking them directly from live birds^23,24^ or carcasses^21,23^, or they can be picked up from nesting sites or floor enclosures when moulted^21,22,25^. Our study used plucked feathers from dead birds and moulted feathers from live birds. DNA concentration from feathers of dead birds varied; for instance, for the wild crows (section "Detection of haemosporidian DNA from feathers"), little to no DNA was recovered possibly due to the advanced degradation that most of them showed (13/15 had an autolysis score of 4 or 5, data not shown), whereas for the birds coming from the Gower Bird Hospital, DNA retrieval rates were considerably higher. This result is likely due to the storage of the samples as GBH bird samples were immediately frozen, whereas the crows remained exposed to the environment for some days, even a week.

Regarding the moulted feathers, they came from two sources, wild Eurasian blackbirds (EB) while being ringed, and captive Sumatran laughingthrushes (SL) whose feathers were collected from the enclosure floor or while birds were in hand. DNA concentration above our preestablished threshold for PCR testing (> 4 ng/µl) was achieved in 11.6% of the EB and 90.4% of the SL. Two factors must be considered concerning these samples. First, the storage conditions, SL feathers were immediately kept at − 20 °C after collection for about two years, while EB feathers were kept at room temperature for up to seven months. Second, the type of feathers employed: for the EB, mostly covert feathers were used, whereas for the SL covert feathers represented only 23.1% of the samples. Considering just covert feathers for the SL, sufficient DNA for PCR was achieved in 66.7% of the extractions compared to 11.3% from the EB; this suggests again, that storage conditions are critical. Nevertheless, we observed a parasite infection out of eight feather pools tested from the EB (12.5%), showing that moulted feathers are a good DNA source for parasite detection. Collection of fallen feathers, recently moulted or while the birds are in hand, should be prioritised over plucking CF of live birds. However, it should be mentioned that not all birds lose feathers as easily as the EB during handling and that for some birds, several feathers may be needed to obtain enough DNA. Likewise, it should be noted that plucking RF from live birds is painful and may produce profuse bleeding as the tips of RF are wide and deeply attached to the skin and underlying tissue, hence it is not recommended. Furthermore, it has been observed that birds with missing rectrices are unable to fly in a straight line^41^, which may impact their fitness. These points should be considered in the planning of systematic surveillance of parasites relaying on the obtaining of feathers.

In agreement with Horváth et al.^21^ and de Volo et al.^22^, RF yielded higher DNA concentrations than CF, possibly due to the comparatively greater amount of tissue contained in the RF^25^. To compensate for the low DNA yields obtained with CF, we pooled them for extraction, which provided enough material for parasite DNA detection. For CF DNA extraction, all researchers agree on using the feather tip. However, debate exists on what part of the RF should be used; some authors have suggested to use the feather tip, as its pulp and follicle cells represent a good DNA source^24,26^; others suggest using the feather calamus, as long as it includes the superior umbilicus, for it contains a small blood clot which provides DNA even in CF^21^. However, the superior umbilicus is not the only part where blood can be found in feathers; de Volo et al.^22^ observed dried blood inside the feather calamus and reported high DNA yields (1500 ng/µl). Likewise, in this study, high DNA concentrations from feathers with observable blood (up to 2131 ng/µl) were recorded, although it should be mentioned that blood was also observed all along the rachis and at the superior rachis. Hence, to obtain the highest DNA yields from feathers, it is recommended to employ the full rachis for CF and small RF, and the rachis contents of large RF.

### Haemosporidian DNA detection in feathers

The performance of genetic analysis with feather-derived DNA is variable across studies. For instance, complete consistency for sex determination between blood and feather extractions^24^, successful use of feather DNA for microsatellite amplification^22^, and a 1.1% misdetections of microsatellites polymorphisms in PCR results^23^ have been reported. In the present study, detection of the most common avian haemosporidians (*Plasmodium* spp., *Leucocytozoon* spp. and *Haemoproteus* spp.) from feather samples was achieved. It was possible to obtain full *cyt b* sequences (479 bp) from feather samples sequenced in both directions; however, as they were compared with shorter one-direction sequences from organ samples they had to be shortened. Noteworthy is that previous protocols employing feather DNA^22–24^ aimed to amplify the bird DNA itself, whilst in this study, we aimed to amplify the haemoparasite’s DNA, which represents a much lower proportion of the sample. For example, a high-intensity infection by these parasites typically represents only 0.5% or more of infected erythrocytes^36^. Likewise, it should be reminded that the PCR method itself is a source of variation, since the repeatability of the nested protocol employed in this work was originally about 83%^32^.

Detection of blood parasites could be difficult even in severely affected birds with high-intensity infections^42^, with one of the reasons probably being the short period during which parasitaemia peaks occur either in the acute phase (generally one to several weeks depending on the species and strain)^2^ or during the relapse, after which, intensity of infection is under one parasite in 1000 erythrocytes and after the latent stage, parasites disappear completely from peripheral blood^2^. The difficulty of detection, added to the associated risks of capturing and handling weakened birds, may lead to the decision of not taking blood samples^6^. Under this scenario, detached feathers could be an invaluable source for fast and reliable infection detection as they could provide similar information to a blood sample (except for specific smear derived information) without posing any danger to the birds. It should be considered that employment of feathers will pose the same advantages or disadvantages as the PCR method chosen for their analysis. Therefore, protocols should be chosen based on the project goals; for instance, detecting multiple haemosporidians at once^32^, increasing the chances of detection and estimating parasitaemia^43^, determining species and lineages^32^, or studying a particular gene of the parasite^44^. In general, molecular methods will increase the chances of parasite detection but they cannot distinguish abortive infections; thus, complementarity with other methods should be considered^45^.

Comparisons of haemosporidian DNA detection between feathers and organs in zoo birds and crows, spending up to one week exposed to the environment, showed a 75% agreement, while the detection of infections between feathers and blood samples from the GHB, that were immediately frozen, showed 100% similarity. As expected, optimally stored feathers are a suitable replacement for blood samples and apparently, they can also provide some information about organ infections. It is possible that the parasite´s life cycle influences the success of detection using feathers and the matching between feather and organ testing results as haemosporidian infections present two main phases, acute and chronic. In the acute phase, a parasitaemia peak appears in peripheral blood for a short period, subsequently parasites migrate to the organs producing considerable damage; if the bird survives, the condition turns chronic presenting light parasitaemia as the parasites remain mostly in the organs for a long time and even for life^2^. Although parasites can be observed in blood or organs at both stages, during the acute phase parasites are predominantly seen in blood as it represents the parasitaemia peak, and during the chronic phase the parasites are predominantly found in organs. As for how that is represented in feathers is not known; feathers can take up to a year to be moulted and in some species, they are replaced twice a year^46^; however, if a feather is damaged or lost prior to the moulting season, it could be replaced at that moment^31^. Consequently, birds have feathers of different ages in their body. If a feather that is analysed for haemosporidian DNA completed its development during or around the parasitaemia peak, then it should represent that acute stage of the infection; whereas if the analysed feather matured long after the parasitaemia peak, then it would likely represent the chronic infection stage. We did not calculate the age of the feathers analysed or infection periods in birds; hence, further studies are required to determine how feathers represent the infection lapse.

Comparison of feather isolated sequences with the corresponding organ or blood sequences (section "Gene sequences match between samples") showed two different scenarios. In the first one, a 99.7–100% match was observed in all feathers (n = 4), and in the second one, one feather showed high similarity to the corresponding blood or organ sequence (98.6–100%), while the other three feathers showed lower similarity (90.5–97.1%). Minor identity differences in feathers sequences from the same bird could be attributed to sequencing errors, whereas major differences could indeed represent different lineages or even species indicating concurrent mixed infections or infections acquired at different times. Further research, including experimental protocols, is required to clarify the diversity of sequences observed from feathers in a single individual.

The GHB feather experiment showed that the detection of blood parasites was enhanced by higher DNA yields. In turn, DNA concentration was increased by samples of heavier birds, the use of remiges and rectrices, and increasing the number of feathers used. RF are preferred, but CF can also provide reliable results; hence, to retrieve sufficient DNA for haemosporidian detection, it is recommended to use one RF or 1–5 CF for birds above 1 kg, 1–2 RF or 10–20 CF feathers for birds between 1 kg and 100 g, and 2–3 RF or 20 CF for birds under 100 g. We recommend that DNA extracts with a concentration above 4.6 ng/µl should be tested expecting a parasite detection probability of 29%. Samples with a DNA concentration above 64.6 ng/µl could be considered optimal as they would provide a 82% probability for parasite detection, which is similar to the replicability of the original protocol (83%)^32^. It should be noted that the feathers used here were kept at optimal conditions when possible and eluted in a low final volume of 40 µl for high DNA concentration. If a low DNA yield is expected, approaches such as pooling feathers^40^ and decreasing the final DNA elution volume^22^ can be used to increase the DNA amount and concentration. Furthermore, if DNA yield after the extraction is considered low for the required test, DNA can be concentrated by an ethanol precipitation protocol, a vacuum centrifuge can be used to reduce liquid volume, or as done in this study, volumes of water and template can be exchanged in the PCR mix. Additionally, PCR cycles can be increased to compensate for the low DNA yield and favour amplification^24^.

## Conclusions

This study demonstrates for the first time that feathers provide non-degraded and sufficient DNA for haemosporidian DNA detection. DNA is protected inside the feathers´ shaft, but the protection is limited and is affected by collection circumstances and the environment. Therefore, optimal sampling conditions and preservation methods should be used for the technique to work reliably. One of the most critical aspects for using feathers as a DNA source is the sample preservation; once the feathers are collected, they should be stored at − 20 °C, or lower temperature, as soon as possible. If feathers are collected in the field, fresher samples are preferred since some degree of degradation will occur from exposure to the environment and although DNA could be extracted from them, it might not have adequate quality for molecular tests. Conservation programs for endangered species and studies of elusive bird species may benefit from this technique since it can be used in a wide range of molecular analyses to gain information about individuals, species, populations, and their pathogens. Likewise, regular screening in captive birds and infection detection in fit and weakened birds could be facilitated; therefore, improving infection control and management without increasing the stress of handling the individuals for conventional sampling.

### Supplementary Information

Supplementary Information.

## Data Availability

All data generated or analysed during this study are included in this published article and its Supplementary information file. Parasite DNA sequences that did not match previously identified lineages were deposited in GenBank (accession numbers OQ418138, OQ418139, OQ435376).

## Abbreviations

CF: Covert feathers
DNA: Deoxyribonucleic acid
EB: Eurasian blackbird
GBH: Gower Bird Hospital
PCR: Polymerase Chain Reaction
PME: Post-mortem Examination
RF: Remiges and rectrices
SL: Sumatran laughingthrush
