# Supplementary material for: A non-invasive feather-based methodology for the detection of blood parasites (Haemosporida)
Source: Sci Rep. 2023 Oct 4;13:16712. doi: 10.1038/s41598-023-43932-y (PMC10550939; doi:10.1038/s41598-023-43932-y)
Supplement: Supplementary file 1 — Supplementary Information. [file 41598_2023_43932_MOESM1_ESM.pdf]

Supplementary Figures

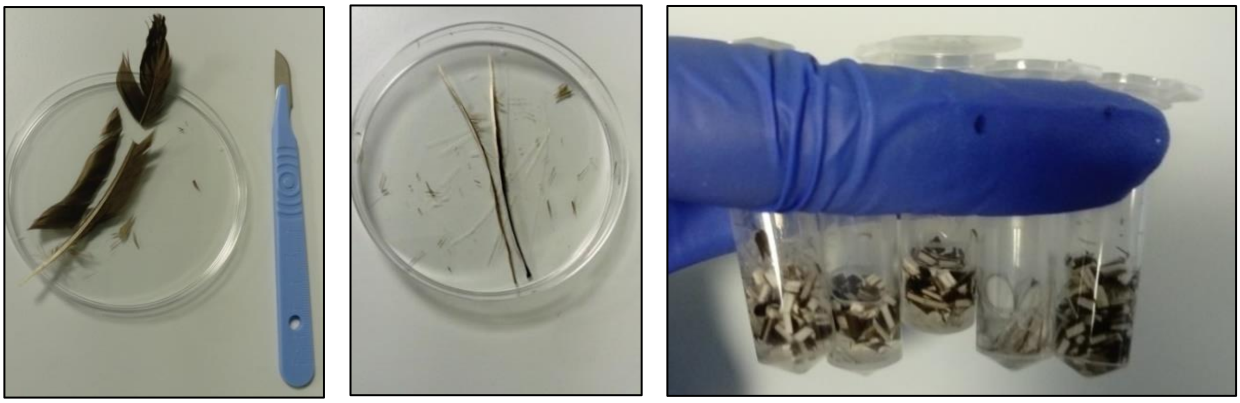

Figure S1. Rachises of flight feathers cut for DNA extraction.

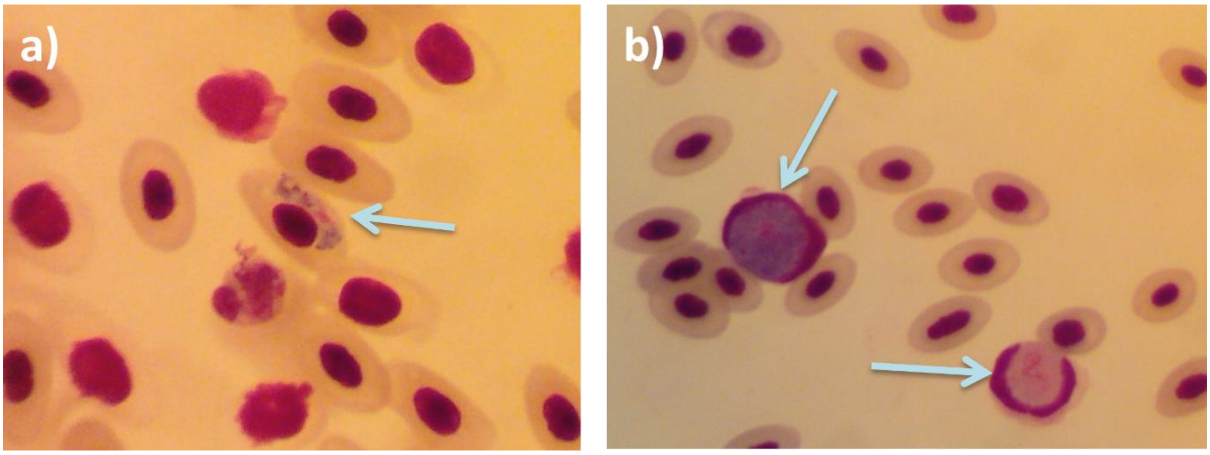

Figure S2. Haemosporidian infections of birds from the Gower Bird Hospital. **a)** *Haemoproteus* spp. infection in a Eurasian blackbird; **b)** *Leucocytozoon* spp. infection in a Carrion crow. Arrows point at gametocytes.

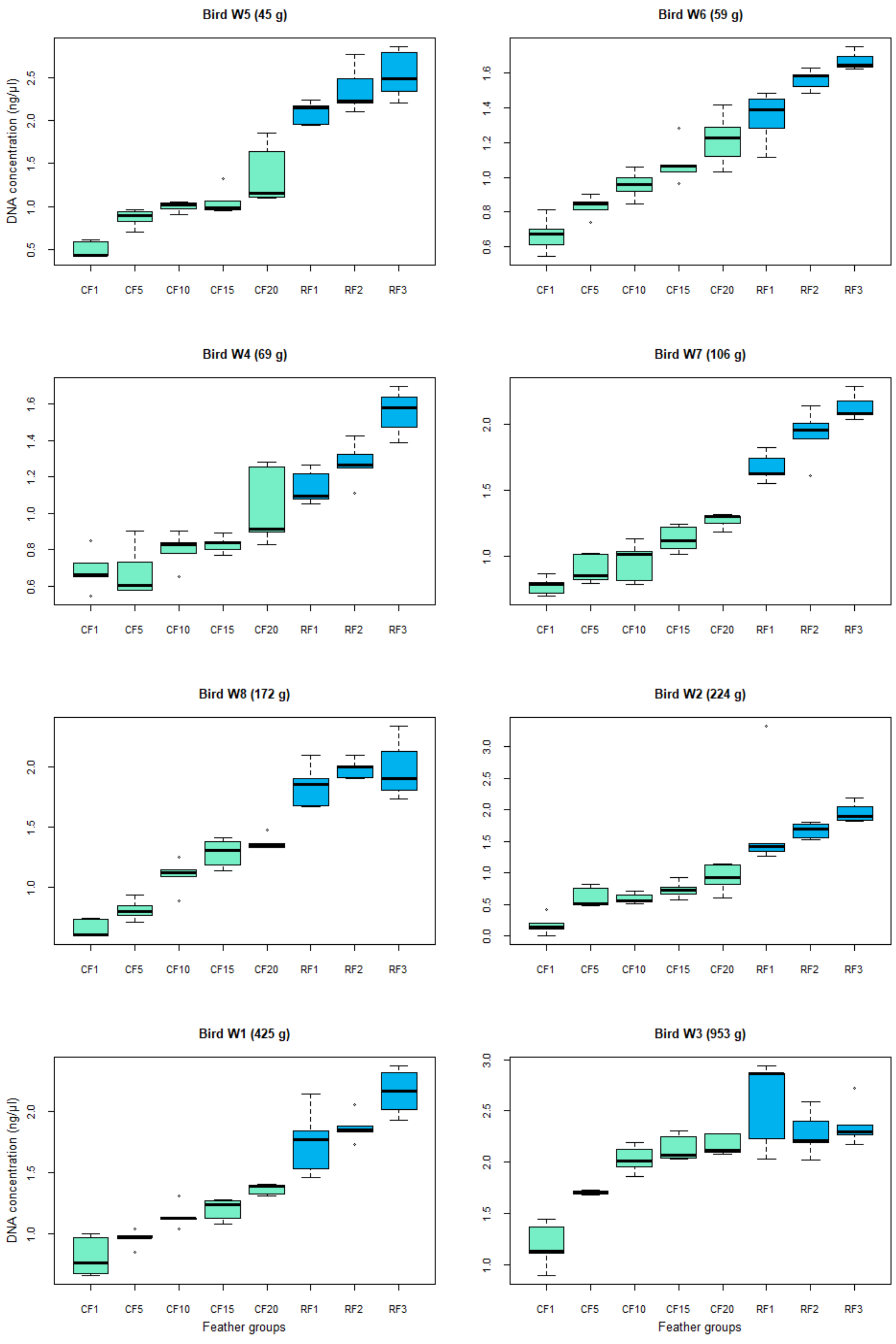

Figure S3. Box plots showing median DNA concentration extracted for each feather group of individual birds. Data for these plots was transformed to $\log_{10}$ (n + 1). Each feather group consisted of five replicates per bird. Birds are ordered from lightest to heaviest.

Supplementary Table

Bird ID	Species	Capture site	Age	Weight (g)	Admission date	Clinical history summary	Blood smear	Blood PCR	Brain PCR
W5	Song thrush (*Turdus philomelos*)	Parkmill	J	45	16/05/2019	Moribund Cold and emaciated	(-)	L	L
W6	Eurasian blackbird (*Turdus merula*)	Llanelli	A	59	03/06/2019	Emaciated Suspected cat attack	H ‡	H	H / L
W4	Song thrush (*Turdus philomelos*)	Winch wen	J	69	24/05/2019	Spinal trauma Cat attack	(-)	H	H / L
W7	Mistle thrush (*Turdus viscivorous*)	Parkmill	A	106	25/04/2019	Moribund Missing rectrices and bald rump	(-)	L	L
W8	Eurasian magpie (*Pica pica*)	Newton	J	172	08/05/2019	Moribund Unable to stand	(-)	L	P / L
W2	Eurasian jackdaw (*Corvus monedula*)	Penllergaer	A	224	14/04/2019	Puncture wound Left wing damaged	(-)	L	L
W1	Carrion crow (*Corvus corone*)	Kidwelly	A	425	07/04/2019	Right humerus broken	L †	nt	H / L
W3	Common raven (*Corvus corax*)	Cwmdu	J	953	05/06/2019	Traumatic injury in caudal spine	(-)	L	L

Table S1. Gower Bird Hospital birds´ data and haemosporidian test results from blood and brain samples. A: Adult; J: Juvenile; P: *Plasmodium* spp.; H: *Haemoproteus* spp.; L: *Leucocytozoon* spp.; ‡: High-intensity infection (> 0.5% of infected erythrocytes); †: Low-intensity infection (< 0.1% of infected erythrocytes); nt: not tested as DNA was not recovered after extraction. Birds are ordered from lightest to heaviest.
